# Health-Related Quality of Life Subdomains in Patients with Parkinson's Disease: The Role of Gender

**DOI:** 10.1155/2018/6532320

**Published:** 2018-08-01

**Authors:** Anja Ophey, Carsten Eggers, Richard Dano, Lars Timmermann, Elke Kalbe

**Affiliations:** ^1^Department of Medical Psychology | Neuropsychology and Gender Studies and Center for Neuropsychological Diagnostics and Intervention (CeNDI), University Hospital Cologne, Kerpener Str. 68, 50937 Cologne, Germany; ^2^Department of Neurology, University Hospital Cologne, Kerpener Str. 62, 50937 Cologne, Germany; ^3^Department of Neurology, University Hospital Gießen Marburg, Baldingerstraße, 35043 Marburg, Germany

## Abstract

The most frequently used instrument to assess health-related quality of life (HrQoL) in Parkinson's disease (PD) is the Parkinson's Disease Questionnaire 39 (PDQ-39). However, both the dimensionality of the eight PDQ-39 subscales and their summary score recently faced criticism. Furthermore, data on disease-related and neuropsychological determinants and the role of gender on HrQoL in PD are inconclusive yet. Therefore, our aim was to reevaluate the PDQ-39 structure and to further explore determinants of HrQoL in PD. 245 PD patients (age: *M* = 69.64, SD = 8.43; 62.9% male; H&Y: Md = 3.00; cognitive assessment with PANDA: *M* = 24.82, SD = 3.57) from the baseline database of the Cologne Parkinson Network were used to reevaluate the dimensionality of the PDQ-39 with a principal component analysis (PCA). Multiple regression analyses were conducted to clarify general and domain-specific relationships between clinical, (neuro)psychological, and sociodemographic variables, gender in particular, and HrQoL. The PCA identified three HrQoL domains: physical-functioning, cognition, and socioemotional HrQoL. Depressive symptoms were identified as the most important determinant of HrQoL across all models. Disease-related HrQoL determinants (UPDRS-III, H&Y stage, and LEDD) were less strong and consistent HrQoL determinants than nonmotor symptoms. Analyses did not reveal a global gender effect; however, female gender was a negative predictor for physical-functioning and socioemotional HrQoL, whereas male gender was a negative predictor for cognition HrQoL. Our analyses suggest the consideration of a reevaluation of the PDQ-39. Only the full understanding of HrQoL, its determinants, and their interrelationships will allow the development of PD intervention strategies focusing on what matters the most for patients' HrQoL. Gender is one relevant variable that should be considered in this context.

## 1. Introduction

Still considered as a paradigmatic movement disorder, Parkinson's disease (PD) is associated with cognitive dysfunctions, depressive symptoms, and a broad spectrum of other nonmotor symptoms (NMS; [1]), as well. Focusing on quality of life in relation to the impact of disease on patients' physical, mental (i.e., emotional and cognitive), and social well-being after diagnosis and treatment, health-related quality of life (HrQoL) has become the preferred concept, when assessing the impact of disease and treatment on the lives of patients [2–4].

To assess HrQoL as a health outcome in PD patients, the Parkinson's Disease Questionnaire 39 (PDQ-39; [5, 6]) was identified as the most appropriate, thoroughly tested, and used questionnaire [7]. The PDQ-39 comprises eight HrQoL subscales [5], commonly summarized by a PDQ-39 summary score [6]. However, recent evidence challenges the validity and interpretability of this summary index. Hagell and Nilsson [8] found that neither Rasch analysis nor confirmatory factor analysis supports for the unidimensionality of the PDQ-39, thus questioning the PDQ-39 summary index. Similarly, the eight-dimensional structure of the PDQ-39 faces criticism to be over-complex and the integration of HrQoL dimensions to a more theoretical framework is demanded [9]. Therefore, one goal of this study was to summarize the eight predefined HrQoL domains to a more meaningful and less complex domain structure based on statistical procedures to reduce data dimensionality. The recent review of Martinez‐Martin [2] emphasized the general importance of a critical evaluation of (Hr)QoL instruments in terms of appropriateness, validity, and psychometric properties.

In a systematic review on HrQoL determinants in PD patients, the influence of NMS to HrQoL, especially depressive symptoms, was highlighted [10]. The negative influence of depression on HrQoL does act not only directly but also indirectly by increasing disability and cognitive dysfunction, which are themselves considered to be negative HrQoL determinants [11–13]. Disease-related HrQoL determinants such as the severity of motor impairment, the overall disease severity, and the levodopa equivalent daily dose (LEDD) were typically less strong and consistent HrQoL determinants than the NMS including depression [10, 14–16]. Concerning demographic variables, heterogeneous results occur, and more studies are necessary to evaluate the influence of age and gender, for example.

Especially for NMS, the existence of gender differences is commonly assumed [17, 18]. However, only a minority of studies was able to identify gender as an independent HrQoL determinant, as the special association of female gender with NMS frequently accounted for observed gender differences in HrQoL [19]. Those findings get even more complex, when gender differences in specific HrQoL domains are taken into account [20, 21]. Emerging evidence for general versus domain-specific relationships in HrQoL determinants is another reason, why an assessment of domain-specific HrQoL determinants might be of special scientific interest: whereas mental health variables were found to be independent HrQoL determinants across all HrQoL dimensions, more domain-specific effects were found in motor-related HrQoL domains [14, 15].

Taken together, the potential of scientific access into the relationships of clinical and sociodemographic HrQoL determinants is far from fully utilized. Thus, the aims of this study were (i) to reevaluate the structure of the PDQ-39 by reducing the number of HrQoL subscales on the basis of the already existing eight-dimensional structure, to further clarify (ii) general and (iii) domain-specific relationships between clinical and sociodemographic variables, gender in particular, and HrQoL of PD patients, and (iv) to explore gender-specific manifestations and moderating effects of depressive symptoms and cognitive impairment onto those relationships. For this purpose, analyses were conducted in use of a large clinical database of the Cologne Parkinson Network (CPN, http://www.koelner-parkinson-netzwerk.uk-koeln.de; [22]).

## 2. Materials and Methods

### 2.1. Participants

For this study, baseline data from the CPN were used. Participants were recruited between January 2012 and July 2015 at the University Hospital of Cologne, Germany, in cooperation with community-based neurologists in greater Cologne. After signing the informed consent form, participants were assessed clinically and neuropsychologically by movement disorder-specialized neurologists or a PD nurse.

To be eligible for enrolment in the CPN study, participants had to be aged 25–85 years, be diagnosed with idiopathic PD according to UK Parkinson's Disease Society Brain Bank diagnostic criteria [23], and have sufficient language ability in German. Exclusion criteria were psychiatric or neurological disorders, severe depressive symptoms operationalized by the Beck's Depression Inventory II [24] (BDI-II; cutoff ≥29), and severe cognitive impairment operationalized by the Parkinson Neuropsychometric Dementia Assessment (PANDA; cutoff ≤14; [25]).

### 2.2. Clinical and Neuropsychological Assessment

#### 2.2.1. Health-Related Quality of Life Assessment

The PDQ-39 [5, 6], a disease-specific, self-evaluative HrQoL instrument, was used to assess HrQoL, with each of the 39 items to be scored on a 5-level scale from 0 (never) to 4 (always). Eight subscale scores and a global HrQoL summary score can be calculated, with all answers being transformed to a 0–100 scale and higher scores representing worse HrQoL. The eight PDQ-39 subscales are mobility, activities of daily living, emotional well-being, stigma, social support, cognitive impairment, communication, and bodily discomfort.

#### 2.2.2. Assessment of Cognition and Nonmotor Symptoms

Global cognitive functioning was assessed with the PANDA (maximum score = 30; [25]), a PD specific cognitive screening tool to assess typical cognitive dysfunctions resulting in mild cognitive impairment in PD (PD-MCI, score 15–17) and dementia (cutoff ≤14). To assess depressive symptoms, the BDI-II (maximum score = 63; [24]), a 21-item self-evaluation questionnaire, was used. A BDI-II score of 9 to 13 reflects minimal depressive symptoms, a score of 14 to 19 mild depressive symptoms, a score of 20 to 28 moderate depressive symptoms, and a score of ≥29 accounts for a severe depressive symptomology. The presence and severity of other NMS was evaluated with the Nonmotor Symptom Scale (NMSS; maximum score = 360; [26]), a 30-item self-evaluation questionnaire.

#### 2.2.3. Clinical Assessment

Disease duration as the time since diagnosis and medication was recorded, as well as the LEDD summarizing the patient's total dopaminergic treatment [27]. Motor impairment was assessed with the motor examination of the Unified Parkinson's Disease Rating Scale Part III (UPDRS-III; [28]) and the Hoehn and Yahr (H&Y; [29]) scale.

### 2.3. Ethical Approval

The study was conducted in compliance with the World Medical Association Declaration of Helsinki (1975). The study protocol was approved by the Ethics Committee of the Medical Faculty of the University of Cologne (Number 11-233) and registered in the German Clinical Trials Register (DRKS00003452).

### 2.4. Statistical Analyses

Statistical analyses were conducted using *R* (http://www.r-project.org). Normal distributions were tested using the Shapiro–Wilk test. Sample characteristics were calculated and compared between genders with Mann–Whitney *U* tests and chi-square tests, each with a significance level of *α* = 0.05. Correlation coefficient *r* was reported as effect size for Mann–Whitney *U* tests.

A higher-order principal component analysis (PCA) was conducted on the eight PDQ-39 subscales, as for example used to develop and validate the PDQ-39 summary score [6, 30]. However, we used Jolliffe's instead of Kaiser's criterion to extract the underlying number of components. Furthermore, to ensure interpretability of the extracted components, and as we assume substantial interrelations between the HrQoL dimensions, we conducted oblique promax rotation on the identified components. Multiple regressions were then used to analyze HrQoL determinants. Baseline HrQoL operationalized by the PDQ-39 total score and the HrQoL domains identified by the PCA, with component scores calculated as mean score of the contributing PDQ-39 subscales, were used as dependent variables in distinct models. Based on the current literature, gender, age, PANDA score, BDI-II score, NMSS score, UPDRS-III score, disease duration, H&Y stage, and LEDD were integrated in the regression models. To further explore relationships between HrQoL determinants, moderated domain-specific HrQoL models extending the domain-specific basic models by gender-specific effects were explored. Significance level for multiple regression analyses was set at *α* = 0.05. Unstandardized (*B*) and standardized (*β*) regression coefficients, *t*-tests for regression coefficients, relative importance of each determinant (*R* ^2^), multiple *R* ^2^, and adjusted *R* ^2^ were reported for each model. Global model fit was tested via *F*-tests. Assumptions for multiple regressions were checked.

## 3. Results

### 3.1. Study Sample Characteristics

Our sample from the baseline data set of the CPN study included 245 patients (37.1% women). Sociodemographic and clinical data of the study sample and gender comparisons are displayed in Table 1. As indicated by Shapiro–Wilk-tests, sample characteristics were not assumed to be normally distributed (*p*s < 0.001). Corresponding *q*-*q* plots for all variables are displayed in Figure S1 (Supplementary Materials). Patients were aged 41 to 86 years (*M* = 69.64, SD = 8.43) with disease duration ranging from just recently to 25 years (*M* = 5.88, SD = 5.73). More than 90% of the patients' PANDA scores fell in the range of normal cognitive functioning (*M* = 24.82, SD = 3.57) and on average, depressive symptoms rated with the BDI-II were minimal to mild (*M* = 12.10, SD = 7.80).

Men and women did not significantly differ in terms of age, reported global HrQoL, severity of NMS, disease duration, and LEDD. The distribution of disease severity according to H&Y stages was comparable between genders. However, PANDA scores were significantly higher for women than for men (*W*=8248, *p*=0.020, *r*=0.15). Additionally, results revealed a nonsignificant tendency for women reporting more severe depressive symptoms than men (BDI-II, *W*=7915, *p*=0.090, *r*=0.11) and men showing more severe motor impairment than women (UPDRS-III, *W*=6090, *p*=0.087, *r*=0.11).

### 3.2. Principal Component Analysis

**Figure 1:**
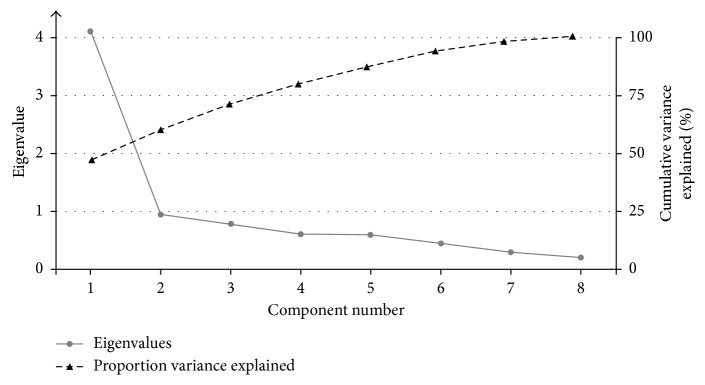
Screeplot of the eigenvalues obtained in the principal component analysis on the eight subscales of the Parkinson's Disease Questionnaire and cumulative variance explained across the eight components.

A PCA was conducted on the eight subscales of the PDQ-39. During an initial analysis without rotation and the maximum number of eight components, only one component had an eigenvalue over Kaiser's criterion of 1 and explained 51% of the variance, which converges well with evidence from earlier studies evaluating the dimensionality of the PDQ-39 [6, 31, 32]. However, three components had eigenvalues over Jolliffe's criterion of 0.70 and in combination explained 73% of the variance, exceeding the 60% criterion [33]. The eigenvalues of each component are displayed in the screeplot of Figure 1, as well as the cumulative variance explained by each of the initial eight components.

As a three-component structure is also compatible with emerging criticism concerning the PDQ-39 structure [8, 9], three components were retained in the subsequent PCA with oblique promax rotation. The PDQ-39 subscales that cluster on the same components suggest that component 1 featuring the PDQ-39 subscales bodily discomfort, mobility, and activities of daily living represents physical-functioning HrQoL (eigenvalue = 2.27, Cronbach's *α* = .81), component 2 featuring the PDQ-39 subscales communication and cognitive impairment represents cognition HrQoL (eigenvalue = 1.91, Cronbach's *α* = 0.76), and component 3 featuring the PDQ-39 subscales emotional well-being, stigma, and social support represents socioemotional HrQoL (eigenvalue = 1.67, Cronbach's *α* = 0.72). Tables S2 and S3 (Supplementary Materials) display the factor loadings of the pattern and structure matrix after oblique promax rotation.

### 3.3. Basic Health-Related Quality of Life Models

Four basic HrQoL models with PDQ-39 summary score, physical-functioning, cognition, and socioemotional scores as dependent variables were calculated. The multiple regression models for global (*g*), physical-functioning (*p*), cognition (*c*), and socioemotional (*s*) HrQoL explained 65.1%, 59.6%, 54.7%, and 46.8% of the total variance (adjusted *R* ^2^ *g*: 63.72%, *p*: 58.0%, *c*: 52.8%, and *s*: 44.7%). Increasing depressive symptoms as indicated by BDI-II scores were a significant negative HrQoL determinant in all four models (*β*_*g*_ = 0.49, *β*_*p*_ = 0.32, *β*_*c*_ = 0.51, and *β*_*s*_ = 0.59). More severe NMS as indicated by NMSS scores were a significant negative HrQoL determinant in three models (*β*_*g*_ = 0.21, *β*_*p*_ = 0.22, and *β*_*c*_ = 0.17). Motor impairment as assessed by the UPDRS-III score occurred as a negative HrQoL determinant in only two models (UPDRS-III: *β*_*g*_ = 0.13 and *β*_*p*_ = 0.25), as well as a higher LEDD (*β*_*g*_ = 0.15 and *β*_*p*_ = 0.18). Female gender was a negative predictor for physical-functioning and socioemotional HrQoL (*β*_*p*_ = −0.11 and *β*_*s*_ = −0.11), whereas male gender was a negative predictor for cognition HrQoL (*β*_*c*_ = 0.19). Less consistent significant HrQoL determinants across all models included a lower cognitive state as indicated by the PANDA total score for cognition HrQoL (*β*_*c*_ = −0.11) and, only marginally significant, younger age for socioemotional HrQoL (*β*_*s*_ = −0.09). Disease duration and H&Y stage were not identified as a significant independent HrQoL determinant in any of the multiple regression models. The number of significant predictors per model varied between two and five. A detailed summary of the multiple regression models is displayed in Table 2.

With regard to the dimension specificity of HrQoL determinants, distributions of relative importance values varied across HrQoL dimensions. On average, depressive symptoms accounted for 46.0% of the total variance explained in HrQoL (*g*: 43.0%, *p*: 25.1%, *c*: 47.5%, and *s*: 68.4%). NMS accounted for around 21.3% of the total variance explained in HrQoL (*g*: 24.6%, *p*: 21.8%, *c*: 23.8%, and *s*: 15.0%). On average, disease-related variables (UPDRS-III, H&Y stage, and LEDD) accounted for 23.8% of the total variance explained in HrQoL (*g*: 26.1%, *p*: 43.6%, *c*: 14.6%, and *s*: 10.7%).

### 3.4. Moderated Domain-Specific Health-Related Quality of Life Models

Multiple regression analyses and model comparisons between hierarchical nested models revealed moderating effects of gender on physical-functioning HrQoL determinants, whereas no evidence for moderating effects was observed in cognition and socioemotional HrQoL. Neither the gender-moderated cognition HrQoL model (adjusted *R* ^2^ = 0.53), *F*(12,222) = 16.86, *p* < 0.001, nor the gender-moderated socioemotional HrQoL model (adjusted *R* ^2^ = 0.44), *F*(17,223) = 11.97, *p* < 0.001, was significantly better than the corresponding basic model (cognition HrQoL Δ*R* ^2^ = 0.02, *F*(8,222) = 1.09, *p*=0.372; socioemotional HrQoL Δ*R* ^2^ = 0.01, *F*(8,223) = 0.48, *p*=0.869).

Originating the basic physical-functioning HrQoL model, the regression model allowing for gender moderations in all potential HrQoL determinants (adjusted *R* ^2^ = 0.60), *F*(17,223) = 22.42, *p* < 0.001, was significantly better than its corresponding basic model, Δ*R* ^2^ = 0.03, *F*(8,223) = 2.67, *p*=0.008. Increasing depressive symptoms (*β* = 0.31) and a higher LEDD (*β* = 0.31) were identified as significant independent negative determinants of physical-functioning HrQoL. Additionally, for both men and women, more severe motor impairment was a significant determinant of physical-functioning HrQoL; however, the relationship was more pronounced for women (*β* = 0.43) than for men (*β* = 0.20). More severe NMS was a significant negative determinant of physical-functioning HrQoL for men only (*β* = 0.04). Note that compared to the basic physical-functioning HrQoL model, gender was not a significant independent determinant of HrQoL anymore. The tendency of women being more affected in the physical-functioning HrQoL domain than men seems to be moderated by the differential influence of the severity of motor symptoms on physical-functioning HrQoL across genders. A detailed summary of the moderated regression model is displayed in Table 3.

## 4. Discussion

The main findings of this study examining the dimensional structure of the PDQ-39 as well as general and domain-specific relationships between clinical and sociodemographic variables, gender in particular, and HrQoL in a cohort of 245 PD patients were as follows: (i) PCA leads to a well interpretable three-component structure of the PDQ-39 with the domains physical-functioning, cognition, and socioemotional HrQoL; (ii) depression and NMS were the strongest and most consistent determinants of global HrQoL and its subdomains; (iii) for disease-related variables and cognition, domain-specific relationships were obtained; and (iv) despite the lack of a gender effect for global HrQoL, domain-specific gender differences and gender-specific manifestations of HrQoL determinants were found.

Further research is needed to evaluate the validity of the proposed three-dimensional HrQoL structure of the PDQ-39 in comparison with the unidimensional and eight-dimensional structure of the PDQ-39, for example, using confirmatory factor analyses and Rasch analyses. The proposed three-dimensional structure already seems more relatable to the domains of the International Classification of Functioning, Disability and Health [34, 35], where the domain of impairment of body functions and structures is relatable to our physical-functioning HrQoL domain and the domain of activity and participation limitations encompasses the cognition and socioemotional HrQoL domain. The proposed three-dimensional structure also mirrors the HrQoL dimensions proposed by Wood-Dauphinee [3] more generally and by Den Oudsten et al. [4] and Martinez‐Martin [2] for PD. However, a limitation of this analysis is its dependency on the eight-dimensional PDQ-39 structure, which is criticized itself [9]. Therefore, we recommend validating the proposed three-dimensional structure from a data-driven point of view that is based on the individual item level.

Depressive symptoms as the most important, independent HrQoL determinant across all regression models support the hypothesis of general relationships between mental health variables and HrQoL [10, 14, 15]. Corroborating results from earlier studies [10, 15], NMS was the second most important determinant of both general HrQoL and its subdomains. However, it must be noted that the NMSS includes a broad range of symptoms, and the total score does not provide information about their nature. Furthermore, the NMSS includes a short assessment of mood and depressive symptoms; thus, a clear distinction of depression and NMS cannot be made with our data.

Other findings of our study point to more specific relationships. Not surprisingly, in line with previous work [14, 15], PD-related HrQoL determinants were strong and consistent HrQoL determinants only in the physical-functioning HrQoL domain. Likewise, global cognitive state was identified as a significant determinant only of cognition HrQoL. However, cognitive impairment in PD is typically associated with poorer HrQoL [12], and the lack of evidence for cognitive state as a determinant of global HrQoL might be due to the skewed range of cognitive abilities of patients in our study.

Our findings revealed a tendency of younger age being a negative determinant of socioemotional HrQoL, which converges well with the hypothesis of younger PD patients having higher HrQoL expectations, facing more difficulties adjusting to disease-related constraints and experiencing more severe psychosocial consequences than older PD patients [36, 37]. Especially the stigma dimension may play a crucial role for reduced socioemotional HrQoL of younger PD patients, whereas opposing effects (i.e., greater disease burden, mobility constraints, and more cognitive impairment for older PD patients) eliminate a global age effect in the other HrQoL domains.

In line with previous literature on domain-specific relationships based on the eight PDQ-39 subscales [20, 21], female gender was a significant negative determinant of physical-functioning and socioemotional HrQoL, whereas male gender was a significant negative determinant of cognition HrQoL, and analyses on global HrQoL revealed no gender effect at all. The moderated regression model of physical-functioning HrQoL further emphasizes the special vulnerability of women concerning their experienced HrQoL through an accentuated negative relationships between motor impairment and physical-functioning HrQoL, possibly due to differential symptom perception and reporting between men and women and the social construction of gender [38].

To the authors' best knowledge, this is the first study evaluating gender-specific manifestations of HrQoL determinants in PD. Although the full spectrum of symptoms should be considered in any patient, knowledge about gender-specific relationships of specific symptoms to HrQoL might sensitize clinicians for symptoms typically reducing HrQoL in men and women and thus to optimize treatment concepts with regard to improving HrQoL. Following the results, the management of depressive symptoms is of outstanding importance in PD interventions for both sexes. Although relevant for all PD patients, the consideration of NMS in the HrQoL context seems especially important for men. Finally, the special vulnerability of men in the cognition HrQoL domain might be due to a close interaction between cognition and communication with job performance and the social construction of male gender [38]. Improving cognitive and communicative abilities, however, might result in an improvement of HrQoL in general and across sexes [39, 40].

Some possible limitations have to be taken into account when interpreting the findings of this study. Even though multiple regression analyses have been the method of choice when evaluating HrQoL determinants [10], they do not take into account the complex interrelationships between HrQoL determinants, as alternative statistical methods such as path analysis and structural modeling could do [41, 42]. Second, despite assessing a wide range of potential variables, some potential HrQoL determinants were not assessed: for example, sociodemographic data on participants' housing situation, marital status, education, and employment status, their quality of sleep, and the nature of NMS and a more detailed assessment of motor complications (e.g., freezing of gate, dyskinesias, and motor fluctuations). Furthermore, assessing symptoms that have to be differentiated from depression, such as apathy and demoralization, could improve the predictive accuracy of HrQoL models [43, 44]. Recently, positive psychological functioning and resilience-related factors have gained attention of HrQoL researchers regarding its protective influence on HrQoL against less modifiable PD symptoms, especially motor ones [14, 45, 46], so that those variables might have accounted for additional variance, most notably in the socioemotional HrQoL domain. Additionally, greater predictive accuracy regarding the relationship of cognition and HrQoL may result from the use of more specific cognitive assessments [47]. Above all, further research is needed to clarify the findings of contributors to HrQoL in a broader sample of PD patients, including individuals with advanced disease (H&Y stage 5) and progressing cognitive impairment (PD-MCI and dementia).

## 5. Conclusions

This study supports and extends previous findings on HrQoL and its determinants in PD patients. A new PDQ-39 component structure dividing HrQoL into a physical-functioning, cognition, and socioemotional domain was proposed. Multiple regression analyses supported evidence for general and domain-specific relationships, emphasize the outstanding importance of depressive symptoms in the management of PD, and highlight scarcely investigated gender-specific manifestations of HrQoL determinants. Only the full understanding of HrQoL determinants and their interrelationships in such an encompassing way will allow the development of new PD intervention strategies that focus on what matters the most for the patients' HrQoL.

## Figures and Tables

**Table 1:** Summary of study sample characteristics and gender comparisons (*n*=245).

	Max. score	*M* (SD) range			*p*
		Total (*n*=245)	Women (*n*=91)	Men (*n*=154)	
Age	—	69.64 (8.43)	70.16 (7.42)	69.34 (8.99)	0.955^b^
		41–86	47–86	41–83	

PDQ-39	100	26.23 (15.08)	26.97 (14.32)	25.79 (15.54)	0.341^b^
		0–79.95	0–79.95	1.67–67.61	
PDQ-39 physical-functioning^a^	100	31.80 (20.17)	33.61 (19.34)	30.73 (20.63)	0.154^b^
		0–86.94	0–79.17	0–86.94	
PDQ-39 cognition^a^	100	26.62 (17.66)	22.77 (15.70)	28.90 (18.40)	**0.017** ^b^
		0–83.34	0–66.66	0–83.34	
PDQ-39 socioemotional^a^	100	20.36 (15.40)	23.02 (16.01)	18.79 (14.85)	**0.032** ^b^
		0–89.58	0–89.58	0–56.25	

PANDA	30	24.82 (3.57)	25.60 (3.09)	24.36 (3.76)	**0.020** ^b^
		14–30	17–30	14–30	
BDI-II	63	12.10 (7.80)	13.03 (7.67)	11.55 (7.84)	0.090^b^
		0–38	0–37	0–38	
NMSS	360	57.31 (31.76)	55.62 (26.15)	58.32 (34.69)	0.987^b^
		6–198	8–122	6–198	

UPDRS-III	108	28.09 (8.94)	26.87 (8.36)	28.81 (9.21)	0.087^b^
		9–65	11–51	9–65	
Disease duration	—	5.88 (5.73)	6.20 (5.76)	5.69 (5.72)	0.426^b^
		0: 25	0: 23	0: 25	
H&Y	5	1: 29	1: 11	1: 18	0.563^c^
		2: 87	2: 34	2: 53	
		3: 106	3: 35	3: 71	
		4: 23	4: 11	4: 12	
LEDD	—	610.07 (408.29)	580.94 (433.05)	627.28 (393.36)	0.204^b^
		0–2065	0–2065	0–1780	

*Note*. Significant gender comparisons appear in bold. BDI-II = Beck's Depression Inventory II; H&Y = Hoehn and Yahr stage; LEDD = levodopa equivalent daily dose; NMSS = Nonmotor Symptom Scale; PANDA = Parkinson Neuropsychometric Dementia Assessment; PDQ-39 = Parkinson's Disease Questionnaire 39; UPDRS-III = Unified Parkinson's Disease Rating Scale Part III. ^a^For a detailed description of PDQ-39 physical-functioning, cognition, and socioemotional component scores, see principal component analysis; ^b^comparison between women and men with the Mann–Whitney *U* test; ^c^comparison between women and men with the chi-square test.

**Table 2:** Results of the multiple regression analyses: basic models.

	Predictor	*B*	SE	*t*	*p*	*R* ^2^
Global health-related quality of life (*n*=242)	Intercept	10.99	3.35	3.28	<0.001^*∗∗∗*^	
	Gender: male^b^	−1.10	1.20	−0.92	0.361	0.00
	Age^a^	−0.00	0.07	−0.00	0.995	0.00
	Disease duration	0.05	0.14	0.35	0.726	0.02
	PANDA^a^	−0.26	0.17	−1.58	0.116	0.01
	BDI-II	0.93	0.09	10.44	<0.001^*∗∗∗*^	0.28
	NMSS^a^	0.09	0.02	4.04	<0.001^*∗∗∗*^	0.16
	UPDRS-III^a^	0.22	0.09	2.29	0.023^*∗*^	0.05
	H&Y	1.72	1.24	1.39	0.167	0.07
	LEDD^a^	0.01	0.00	2.81	0.005^*∗∗*^	0.05
		*F*(9,232) = 48.02, *p* < 0.001			Multiple *R* ^2^	0.65
					Adjusted *R* ^2^	0.64

Physical-functioning health-related quality of life (*n*=241)	Intercept	16.99	4.95	3.43	<0.001^*∗∗∗*^	0.01
	Gender: male^b^	−4.67	1.79	−2.61	0.010^*∗∗*^	0.01
	Age^a^	0.03	0.10	0.26	0.798	0.01
	Disease duration	−0.04	0.22	−0.17	0.868	0.03
	PANDA^a^	−0.09	0.25	−0.38	0.706	0.01
	BDI-II	0.81	0.13	6.23	<0.001^*∗∗∗*^	0.15
	NMSS^a^	0.13	0.03	3.90	<0.001^*∗∗∗*^	0.13
	UPDRS-III^a^	0.56	0.14	3.98	<0.001^*∗∗∗*^	0.10
	H&Y	3.02	1.84	1.64	0.101	0.10
	LEDD^a^	0.01	0.00	3.19	0.002^*∗∗*^	0.06
		*F*(9,231) = 37.80, *p* < 0.001			Multiple *R* ^2^	0.60
					Adjusted *R* ^2^	0.58

Cognition health-related quality of life (*n*=240)	Intercept	0.99	4.63	0.21	0.831	
	Gender: Male^b^	6.97	1.66	4.21	<0.001^*∗∗∗*^	0.03
	Age^a^	0.14	0.09	1.52	0.131	0.01
	Disease duration	0.16	0.20	0.82	0.416	0.01
	PANDA^a^	−0.55	0.23	−2.40	0.017^*∗*^	0.02
	BDI-II	1.16	0.13	9.07	<0.001^*∗∗∗*^	0.26
	NMSS^a^	0.09	0.03	2.86	0.005^*∗∗*^	0.13
	UPDRS-III^a^	−0.13	0.13	−1.00	0.318	0.02
	H&Y	2.80	1.70	1.65	0.100	0.04
	LEDD^a^	0.00	0.00	0.75	0.453	0.02
		*F*(9,230) = 53.68, *p* < 0.001			Multiple *R* ^2^	0.55
					Adjusted *R* ^2^	0.53

Socioemotional health-related quality of life (*n*=241)	Intercept	9.56	4.18	2.29	0.023^*∗*^	
	Gender: Male^b^	−3.36	1.51	−2.23	0.027^*∗*^	0.01
	Age^a^	−0.17	0.09	−1.94	0.053	0.01
	Disease duration	0.12	0.17	0.66	0.510	0.01
	PANDA^a^	−0.25	0.21	−1.23	0.220	0.01
	BDI-II	1.13	0.11	10.14	<0.001^*∗∗∗*^	0.32
	NMSS^a^	0.02	0.03	0.72	0.471	0.07
	UPDRS-III^a^	0.20	0.12	1.73	0.085	0.02
	H&Y	−0.43	1.53	−0.28	0.779	0.02
	LEDD^a^	0.00	0.00	0.83	0.407	0.01
		*F*(9,231) = 22.58, *p* < 0.001			Multiple *R* ^2^	0.47
					Adjusted *R* ^2^	0.45

*Note*. Parkinson's Disease Questionnaire 39 (PDQ-39) total score and the PDQ-39 component scores physical-functioning, cognition, and socioemotional HrQoL (as revealed by the principal component analysis) were used as dependent variables. BDI-II = Beck's Depression Inventory II; H&Y = Hoehn and Yahr stage; LEDD = levodopa equivalent daily dose; NMSS = Nonmotor Symptom Scale; PANDA = Parkinson Neuropsychometric Dementia Assessment; UPDRS-III = Unified Parkinson's Disease Rating Scale Part III. ^a^Variable was mean-centered; ^b^gender was dummy coded with female gender as the baseline group; ^*∗*^ *p* ≤ 0.05;  ^*∗∗*^ *p* ≤ 0.01;  ^*∗∗∗*^ *p* ≤ 0.001.

**Table 3:** Results of the gender-moderated multiple regression analysis on physical-functioning health-related quality of life (*n*=241).

	*B*	SE	*t*	*p*
Intercept	15.38	8.38	1.84	0.068
Gender: male^b^	0.01	10.04	<0.01	0.999
Age^a^	0.01	0.18	0.03	0.974
Age gender	0.09	0.22	0.42	0.678
Disease duration	−0.27	0.39	−0.70	0.483
Disease duration gender	0.17	0.47	0.36	0.719
PANDA^a^	0.18	0.47	0.39	0.696
PANDA gender	−0.36	0.55	−0.66	0.511
BDI-II	0.78	0.20	3.87	<0.001^*∗∗∗*^
BDI-II gender	0.01	0.26	0.03	0.978
NMSS^a^	0.01	0.06	0.10	0.920
NMSS gender	0.20	0.07	2.75	0.006^*∗∗*^
UPDRS-III^a^	0.95	0.26	3.69	<0.001^*∗∗∗*^
UPDRS-III gender	−0.59	0.31	−1.92	0.046^*∗*^
H&Y	3.98	3.09	1.29	0.200
H&Y gender	−2.06	3.81	−0.54	0.589
LEDD^a^	0.01	0.01	3.23	0.001^*∗∗*^
LEDD gender	−0.01	0.01	−1.55	0.122
		Multiple *R* ^2^		0.63
		Adjusted *R* ^2^		0.60

*Note*. BDI-II = Beck's Depression Inventory II; LEDD = levodopa equivalent daily dose; NMSS = Nonmotor Symptom Scale; UPDRS-III = Unified Parkinson's Disease Rating Scale Part III. ^a^Gender was dummy coded with female gender as the baseline group; ^b^variable was mean-centered; ^*∗*^ *p* ≤ 0.05;  ^*∗∗*^ *p* ≤ 0.01;  ^*∗∗∗*^ *p* ≤ 0.001.

## Data Availability

The data used to support the findings of this study are available from the corresponding author upon request.

## Supplementary Materials

Supplementary Materials Figure S1 displays q-q plots for all variables used in this study. Tables S2 and S3 extend the reported data of the PCA on the eight original subscales of the PDQ-39 and display the factor loadings of the pattern and structure matrix after oblique promax rotation.
