# Supplementary material for: Health-Related Quality of Life Subdomains in Patients with Parkinson's Disease: The Role of Gender
Source: Parkinsons Dis. 2018 Aug 1;2018:6532320. doi: 10.1155/2018/6532320 (PMC6093079; doi:10.1155/2018/6532320)
Supplement: Supplementary Materials — Figure S1 displays q-q plots for all variables used in this study. Tables S2 and S3 extend the reported data of the PCA on the eight original subscales of the PDQ-39 and display the factor loadings of the pattern and structure matrix after oblique promax rotation. [file 6532320.f1.pdf]

Supplementary Material

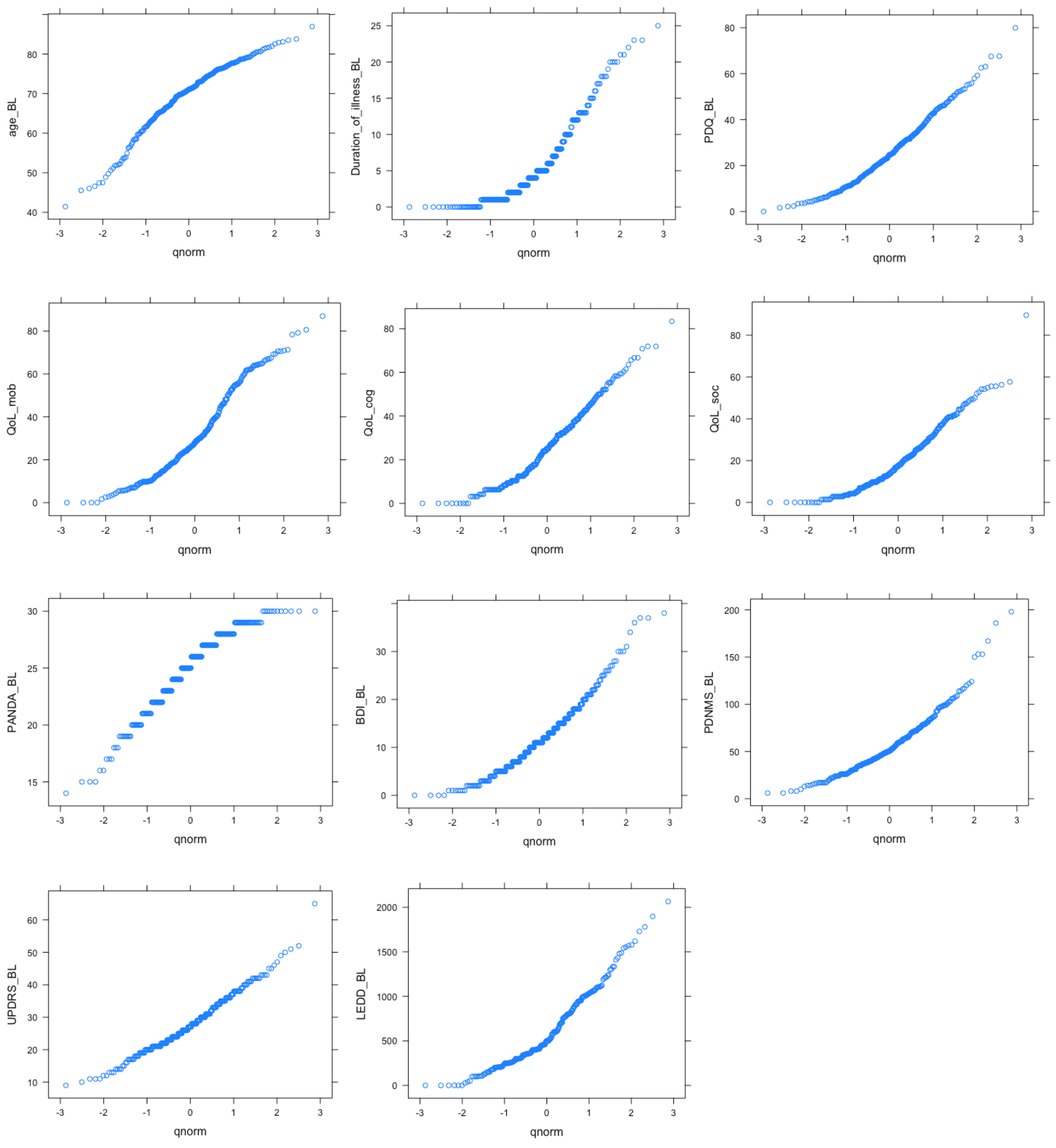

Figure S1. Q-Q plots for the normality tests of all variables used in the study.

Table S2

Summary of the Factor Analysis on Parkinson's Disease Questionnaire 39 Dimensions: Pattern Matrix (N = 245) after Oblique Promax Rotation

	Promax Rotated Factor Loadings			
PDQ-39 Dimension	Component 1	Component 2	Component 3	h^2
Bodily Discomfort	**.98**	-.17	-.10	.71
Mobility	**.80**	.12	.03	.80
Activities of Daily Living	**.54**	.45	-.09	.70
Communication	-.13	**.96**	.04	.83
Cognitive Impairment	.10	**.77**	.00	.69
Emotional Well-Being	.45	.07	**.46**	.71
Stigma	-.26	.18	**.89**	.73
Social Support	.19	-.20	**.79**	.67
Eigenvalue	2.27	1.91	1.67	
% Variance Explained	28	24	21	∑ 73
Cronbach's α	.81	.76	.72	

Note. Factor loadings > .45 appear in bold. h^2 = communality. PDQ-39 = Parkinson's Disease Questionnaire 39.

Table S3

Structure Matrix of the Factor Analysis on Parkinson's Disease Questionnaire 39 Dimensions (N = 245) after Oblique Promax Rotation

	(Pattern Matrix) * (Correlation Matrix of Factors)		
PDQ-39 Dimension	Component 1	Component 2	Component 3
Bodily Discomfort	**.83**	.36	.34
Mobility	**.88**	**.60**	**.50**
Activities of Daily Living	**.76**	**.73**	.39
Communication	**.46**	**.91**	.40
Cognitive Impairment	**.56**	**.83**	.40
Emotional Well-Being	**.73**	**.54**	**.73**
Stigma	.31	.42	**.83**
Social Support	**.48**	.26	**.80**

Note. Factor loadings > .45 appear in bold. PDQ-39 = Parkinson's Disease Questionnaire 39.
